# ω-3 fatty acid-enriched parenteral nutrition shortens hospital stay in acute variceal bleeding cirrhotic patients

**DOI:** 10.1097/MD.0000000000029128

**Published:** 2022-04-08

**Authors:** Seong-Jung Kim, In Ae Chun, Ju-Yeon Cho, Jun Hyung Lee, Jun Lee, Young-Dae Kim, Chan-Guk Park

**Affiliations:** a Department of Internal Medicine, Chosun University Hospital, Gwang-Ju, Korea; b Clinical Nutrition, Chosun University Hospital, Gwang-Ju, Korea.

**Keywords:** hospital stay, liver cirrhosis, omega-3 fatty acid, parenteral nutrition, variceal bleeding

## Abstract

Acute variceal bleeding, a crucial complication of liver cirrhosis requires high energy expenditures but gastrointestinal bleeding limits enteral feeding in the acute stage. We investigated the safety and efficacy of ω-3 fatty acid-enriched parenteral nutrition in acute variceal bleeding patients.

In this retrospective study, a total of 208 cirrhotic patients with acute variceal bleeding who underwent parenteral nutrition in the absence of enteral nutrition were enrolled. Among the patients, 86 patients received ω-3 fatty-acid-enriched parenteral nutrition. The primary endpoint was to evaluate the duration of hospital stay and the presence of clinical complications of liver cirrhosis.

The mean age of the patients enrolled was 54.9 years-old and 185 patients (88.9%) were male. The cause of liver cirrhosis, Child-Pugh score and comorbidities were statistically not different. Patients with ω-3 enriched parenteral nutrition had a significantly lower systolic blood pressure and total bilirubin levels. The difference in the in-hospital mortality (*P* = .813) or rate of complications (*P* = .880) was not statistically significant. The duration of hospital stay was significantly shorter in the patients who underwent ω-3 fatty acid-enriched parenteral nutrition (10.7 ± 7.3 vs 7.9 ± 4.2 days, *P* = .001).

In liver cirrhosis patients with acute variceal bleeding, ω-3 fatty acid-enriched parenteral nutrition significantly decreased the length of hospital stay. Further prospective studies to consolidate these findings are warranted.

## 1 Introduction

Liver cirrhosis is prevalent worldwide and can be a consequence of different causes, such as alcohol consumption, hepatitis B or C infection, obesity, nonalcoholic fatty liver disease, autoimmune diseases, cholestatic diseases, and iron or copper overload.^[1]^ Liver cirrhosis develops after a long period of inflammation that results in replacement of the healthy liver parenchyma with fibrotic tissue and regenerative nodules, leading to portal hypertension.^[2]^ In cirrhotic patients, an increase in hepatic-venous pressure gradient primarily from an increase in intrahepatic resistance leads to development of portosystemic collaterals.^[3]^ One of the most important portosystemic collateral is the esophageal and gastric varices that form at the distal esophagus and proximal stomach. Mucosal rupture of the esophageal/gastric varices leads to profound bleeding with a 6-week mortality rate of 15% to 50%.^[1,4]^ Endoscopic variceal ligation (EVL) to control the acute bleeding involves suctioning of the varix into a round tip at the end of the endoscope and applying rubber bands around the varix.^[5,6]^ After control of the acute variceal rupture, there has been recommendations to avoid oral and/or enteral nutrition in these cirrhotic patients for 48 hours.^[7]^

However, cirrhotic patients exhibit hepatic glycogen depletion and resort to protein catabolism for gluconeogenesis much earlier than noncirrhotic patients.^[8]^ Malnutrition in these patients is associated with a higher prevalence of ascites, hepatorenal syndrome, longer hospital-stay, higher costs, and increased mortality.^[9]^ In acute variceal bleeding cirrhotic patients, greater energy intake is necessary and the importance of adequate nutrition is paramount but easily overlooked.^[10]^ In considering the energy composition of the nutrition supplementation, omega-3 polyunsaturated fatty acids (PUFA) are now well known for their anti-inflammatory and immunomodulatory properties.^[11]^ A recent meta-analysis reported decreased infection rates and shortened hospital stay in intensive care unit patients receiving ω-3 PUFA-based parenteral nutrition (PN).^[12]^

In the present study, the safety and efficacy of ω-3 enriched PUFA-based PN in acute variceal bleeding cirrhotic patients compared to patients with earlier generations of PN was determined. To the best of our knowledge, this is the first study to evaluate the clinical outcome and safety of ω-3 enriched PUFA based PN in liver cirrhosis patients with acute variceal bleeding.

## 2 Methods

### 2.1 Patients

**Figure 1:**
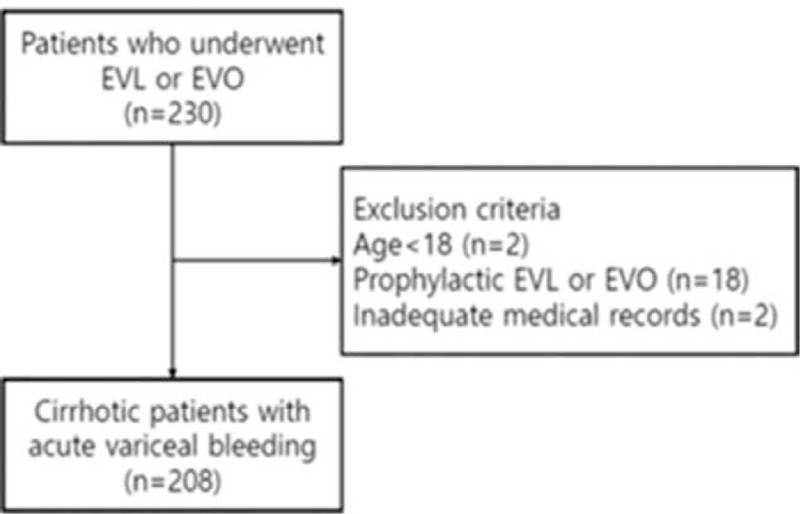
Flow diagram of enrolled patients in the study. A total of 208 cirrhotic patients with acute variceal bleeding who underwent endoscopic treatment that had PN in the absence of enteral nutrition were enrolled. EVL = endoscopic variceal ligation, EVO = endoscopic variceal obturation.

Cirrhotic patients with acute variceal bleeding who underwent PN in the absence of enteral nutrition at Chosun University Hospital between January 2013 and December 2017 were evaluated in this retrospective study. A total of 230 cirrhotic patients with acute variceal bleeding underwent endoscopic treatment such as EVL or endoscopic variceal obliteration (EVO) during the period and 208 patients were enrolled in the study after exclusion based on the following criteria; patients younger than 18 years of age, prophylactic EVL or EVO was performed, and inadequate medical records (Fig. 1). The primary endpoint was to evaluate the safety and efficacy of PN containing fatty acids with ω-6 to ω-3 ratio of 2.1 to 1. The duration of hospital-stay and the development of clinical complications of liver cirrhosis and mortality was evaluated. This study was conducted in accordance with the guidelines of the Declaration of Helsinki and principles of Good Clinical Practice. The patient consent was waived as for the retrospective design of the study and approved by the local institutional review board (Chosun University Hospital 2018-01-015).

### 2.2 Management of acute variceal bleeding

Although the study is retrospective, the general management of acute variceal bleeding at the study site was as follows. Cirrhotic patients with variceal bleeding underwent EVL or EVO after confirmed variceal bleeding through emergency esophagogastroduodenoscopy within 12 hours. The patients were administered 3 days of terlipressin and 5 to 7 days of 3^rd^ generation cephalosporin unless complications needing additional treatment was necessary. The treatments for the liver related complications included liver transplantation (2 patients) and transjugular intrahepatic portosystemic shunt (1 patient). In 4 patients, progression to hepatorenal syndrome necessitated the use of albumin and terlipressin up to 14 days.

### 2.3 Supplement of parenteral nutrition

Patients supplied with parenteral nutrition that underwent endoscopic procedures to treat variceal bleeding were included. The patients were divided into control vs ω-3 enriched groups. The control group received parenteral nutrition containing fatty acids with ω-6 to ω-3 ratio greater than 2.1 to 1. The ω-3 enriched group received parenteral nutrition containing fatty acids with ω-6 to ω-3 ratio of 2.1 to 1.

### 2.4 Assessment of baseline laboratory measurements and clinical outcome

Data on baseline laboratory measurements and clinical outcome were collected through the medical record review. The patients were evaluated for age, gender, etiology of liver cirrhosis, systolic blood pressure, diastolic blood pressure, pulse rate, and laboratory tests such as white blood cell count, hemoglobin, platelet count, prothrombin time-international normalized ratio, aspartate transaminase, alanine transaminase, total bilirubin (T-bil), serum albumin, blood urea nitrogen, creatinine, serum sodium, erythrocyte sedimentation rate (ESR), and C-reactive protein. The laboratory results were evaluated at baseline and on day 5 of hospital day or if absent, the closest result available. Child-Pugh class consisting of scores based on values of serum T-bil, albumin, prothrombin time-international normalized ratio, state of ascites and hepatic encephalopathy was evaluated. The in hospital-stay, development of complications including rebleeding, spontaneous bacterial peritonitis, hepatic encephalopathy, sepsis, hepatic failure, and in hospital mortality was assessed.

### 2.5 Statistical analysis

Continuous variables are presented as mean ± standard deviation and a median value with range according to parametric and nonparametric distributions, respectively. The variables are compared parametrically using the Student *t* test or nonparametrically using the Mann–Whitney *U* test. Categorical variables are presented as frequencies with percentages and compared by chi-square or Fisher exact test. A multiple linear regression was used to evaluate the factors correlating to the duration of in-hospital stay. A two-sided *P* < .05 was considered statistically significant. All statistical analyses were performed using SPSS version 25.0 (IBM Co., Armonk, NY).

## 3 Results

### 3.1 Baseline demographic findings

The baseline characteristics of the enrolled patients are shown in Table 1. A total of 208 cirrhotic patients with acute variceal bleeding who underwent esophageal varices band ligation that had PN in the absence of enteral nutrition were enrolled. One hundred twenty-two patients had PN containing fatty acids with ω-6 to ω-3 ratio greater than 2.1 to 1 (control) while 86 patients had PN containing fatty acids with ω-6 to ω-3 ratio of 2.1 to 1 (ω-3 enriched). The mean age of the patients was 54.9 years, and most of the patients were male (88.9%). Concomitant comorbidities of hypertension, diabetes mellitus, end-stage renal disease, hepatocellular carcinoma was noticed in 20 (9.6%), 47 (22.6%), 4 (1.9%), and 34 (16.3%) patients, respectively. The predominant cause of liver cirrhosis was hepatitis B virus in 146 (70.2%) patients, followed by hepatitis C virus, combined hepatitis B virus and hepatitis C virus, autoimmune, alcohol and unknown in 40 (19.2%), 11 (5.3%), 5 (2.4%), 4 (1.9%), and 2 (1.0%) patients, respectively. The mean systolic and diastolic blood pressure were 98.3 mm Hg and 59.7 mm Hg. The Child-Pugh class of the patients were class A in 38 (18.3%), class B in 116 (55.8%) and class C in 54 (26.0%) patients. The duration of infusion of PN were 3.28 days in the ω-3 enriched PUFA-based PN group and 3.02 days in the control group.

**Table 1:** Baseline characteristics.

	All patients	Omega-3 (–)	Omega-3 (+)	*P* value
N	208	122	86	
Age	54.9 ± 10.5	55.0 ± 10.4	54.8 ± 10.6	.889
Sex				.071
Male	185 (88.9)	113 (92.6)	72 (83.7)	
Female	23 (11.1)	9 (7.4)	14 (16.3)	
Comorbidities				
HTN	20 (9.6)	12 (9.8)	8 (9.3)	1
DM	47 (22.6)	26 (21.3)	21 (24.4)	.617
ESRD	4 (1.9)	3 (2.4)	1 (1.2)	.673
HCC	34 (16.3)	24 (19.7)	10 (11.6)	.133
Cause of liver cirrhosis				.411
Alcohol	4 (1.9)	3 (2.5)	1 (1.2)	
HBV	146 (70.2)	86 (70.5)	60 (69.8)	
HCV	40 (19.2)	24 (19.7)	16 (18.6)	
HBV + HCV	11 (5.3)	5 (4.1)	6 (7.0)	
Autoimmune	5 (2.4)	4 (3.3)	1 (1.2)	
Unknown	2 (1.0)	.	2 (2.3)	
sBP	98.3 ± 23.5	101.6 ± 23.3	93.5 ± 23.1	.013
dBP	59.7 ± 15.8	61.2 ± 16.0	57.4 ± 15.3	.089
Pulse rate	99.0 ± 21.1	97.4 ± 20.3	101.4 ± 22.0	.187
Child-Pugh class				.633
A	38 (18.3)	20 (16.4)	18 (20.9)	
B	116 (55.8)	71 (58.2)	45 (52.3)	
C	54 (26.0)	31 (25.4)	23 (26.7)	
MELD score	15.76 ± 6.89	16.07 ± 7.00	15.31 ± 6.74	.432

dBP = diastolic blood pressure, DM = diabetes mellitus, ESRD = end stage renal disease, HBV = hepatitis B virus, HCC = hepatocellular carcinoma, HCV = hepatitis C virus, HTN = hypertension, sBP = systolic blood pressure.

Between the control and ω-3 enriched group, only the systolic blood pressure showed a significant difference. The ω-3 enriched group had a significantly lower systolic blood pressure of 93.5 mm Hg compared to 101.6 mm Hg in the control group (*P* = .013). There was no significant difference in the number of patients in each Child Pugh class between the control and the ω-3 enriched group (*P* = .633).

### 3.2 Laboratory findings – baseline and follow up

There were no major differences in the baseline characteristics except for T-bil and ESR between the control and ω-3 enriched group. The control group had a significantly higher total bilirubin of 3.8 mg/dL compared to 2.8 mg/dL in the ω-3 enriched group (*P* = .023). The control group had a significantly higher mean ESR of 14.2 mm/h compared to 9.1 mm/h in the ω-3 enriched group (*P* = .027).

The post laboratory findings showed significant difference in the T-bil, albumin, and ESR between the control and ω-3 enriched group. The control group had a significantly higher total bilirubin of 3.4 mg/dL compared to 2.1 mg/dL in the ω-3 enriched group (*P* = .046). Albumin levels were 2.8 mg/dL in the control group and 3.0 mg/dL in the ω-3 enriched group. The difference in the albumin levels between the control and ω-3 enriched group was statistically significant (*P* = .009). The control group had a significantly higher mean ESR of 17.9 mm/h compared to 9.1 mm/h in the ω-3 enriched group (*P* = .027).

The difference in the baseline laboratory values from the post laboratory values were evaluated to determine the changes in the laboratory results between the control and ω-3 enriched groups during the admission period. There was a significantly greater decrease in white blood cell counts in the control group (4336.7 ± 4853) compared to the ω-3 enriched group (2982.9 ± 3816) (*P* = .032). Although statistically insignificant, a trend for a greater increase in albumin levels in the ω-3 enriched group (0.19 ± 0.5) compared to the control group (0.05 ± 0.4) was noticed (*P* = .050) (Table 2).

**Table 2:** Laboratory differences during treatment.

	Omega-3 (–) (n = 122)			Omega-3 (+) (n = 86)			*P* value^∗^	*P* value^†^	*P* value^‡^
	Baseline	Post	Delta	Baseline	Post	Delta	Baseline	Post	Delta
WBC	9970.5 ± 6327.1	5633.7 ± 3299.5	−4336.7 ± 4853	8687.3 ± 3736.5	5704.4 ± 3260.1	−2982.9 ± 3816	.068	.878	.032
Hgb	8.7 ± 2.6	8.9 ± 1.8	0.18 ± 1.8	8.1 ± 2.5	8.6 ± 1.2	0.50 ± 2.2	.091	.210	.274
Platelet	124.1 ± 61.5	98.2 ± 48.2	−25.87 ± 55.4	117.2 ± 62.0	96.1 ± 56.2	−21.1 ± 52.2	.432	.773	.533
PT (INR)	1.60 ± 0.5	1.59 ± 2.1	−0.004 ± 2.1	1.58 ± 0.4	1.39 ± 0.5	−0.17 ± 0.4	.702	.311	.379
AST	178.7 ± 450.7	152.5 ± 671.7	−26.17 ± 786.7	91.4 ± 103.6	115.3 ± 197.2	23.9 ± 160.6	.080	.564	.496
ALT	53.8 ± 77.9	81.1 ± 274.7	27.31 ± 275.1	39.3 ± 39.9	94.7 ± 376.8	55.4 ± 367.9	.116	.775	.550
T-bil	3.8 ± 5.4	3.4 ± 5.3	−0.44 ± 2.5	2.4 ± 2.9	2.1 ± 3.1	−0.26 ± 1.6	.023	.046	.525
Albumin	2.8 ± 0.6	2.8 ± 0.5	0.05 ± 0.4	2.8 ± 0.7	3.0 ± 0.5	0.19 ± 0.5	.693	.009	.050
BUN	28.6 ± 20.7	18.2 ± 19.3	−10.37 ± 19.7	30.4 ± 21.9	22.6 ± 18.7	−7.80 ± 20.6	.556	.105	.369
Cr	1.1 ± 0.8	0.8 ± 0.5	−0.24 ± 0.6	1.1 ± 1.0	0.9 ± 0.9	−0.89 ± 0.8	.738	.305	.167
ESR	14.2 ± 17.9	17.9 ± 18.5	3.72 ± 12.7	9.1 ± 12.8	12.2 ± 14.3	3.09 ± 12.6	.027	.014	.722
CRP	1.2 ± 1.5	1.3 ± 1.5	0.09 ± 1.3	1.3 ± 2.9	1.5 ± 2.4	0.16 ± 2.7	.777	.582	.849

ALT = alanine transaminase, AST = aspartate transaminase, BUN = blood urea nitrogen, Cr = creatinine, CRP = C-reactive protein, ESR = erythrocyte sedimentation rate, Hgb = hemoglobin, INR = international normalized ratio, PT = prothrombin time, T-bil = total bilirubin, WBC = white blood cells.

∗ The difference in the baseline laboratory findings between the ω-3 negative group and the ω-3 positive group.

† The difference in the post laboratory findings between the ω-3 negative group and the ω-3 positive group.

‡ The difference in the (Post laboratory findings-Baseline laboratory findings) between the ω-3 negative group and the ω-3 positive group.

### 3.3 In-hospital morbidity and mortality

The mean in-hospital stay in all the patients was 9.6 days. Patients with any morbidity during the admission period were reviewed. Rebleeding, spontaneous bacterial peritonitis, hepatic encephalopathy, sepsis, hepatic failure, and other causes were noticed in 13 (6.3%), 5 (2.4%), 4 (1.9%), 11 (5.3%), 18 (8.7%), and 10 (4.8%) patients, respectively. In hospital mortality in the acute variceal bleeding liver cirrhosis patients was noted in 20 (9.6%) patients.

**Figure 2:**
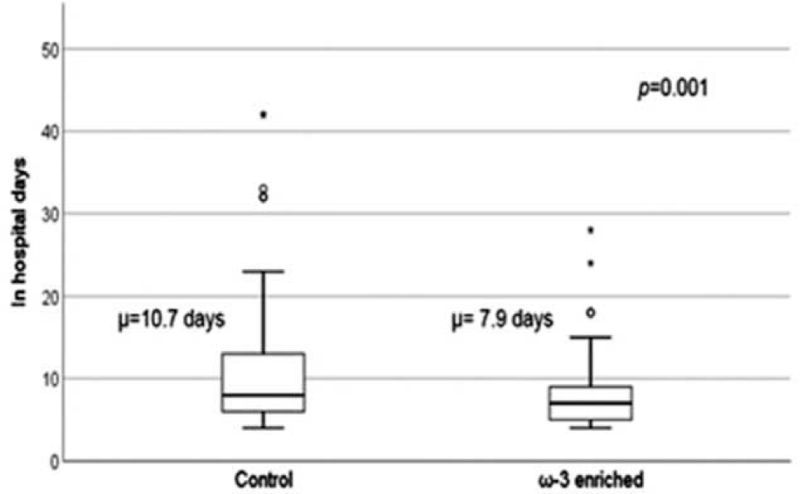
Shortened in-hospital days in the ω-3 enriched parenteral nutrition group. The in-hospital stay was 7.9 d in the ω-3 enriched group compared to the 10.7 d in the control group.

The in-hospital stay was 7.9 days in the ω-3 enriched group compared to the 10.7 days in the control group (Fig. 2). The difference in the in-hospital stay was statistically significant (*P* = .001). The complication rate related to hepatic failure was not significantly different in the 2 groups. However, other complications including influenza, acute kidney injury, pleural effusion, and paroxysmal atrial fibrillation occurred significantly higher in the control group (*P* = .049). There was no difference in the in-hospital mortality between the control and the ω-3 enriched group (Table 3).

**Table 3:** In hospital morbidity and mortality.

	All patients	Omega-3 (–)	Omega-3 (+)	*P* value
N	208	122	86	
In hospital stay	9.6 ± 6.3	10.7 ± 7.3	7.9 ± 4.2	.001
Complications	61 (29.3)	34 (27.9)	27 (31.4)	.643
Rebleeding	13 (6.3)	5 (4.1)	9 (9.3)	.152
SBP	5 (2.4)	3 (2.5)	2 (2.3)	1.000
Hepatic encephalopathy	4 (1.9)	2 (1.6)	2 (2.3)	1.000
Sepsis	11 (5.3)	5 (4.1)	6 (7.0)	.368
Hepatic failure	18 (8.7)	10 (8.2)	8 (9.3)	.806
Others	10 (4.8)	9 (7.4)	1 (1.2)	.049
In hospital mortality	20 (9.6)	11 (9.0)	9 (10.5)	.813

SBP = spontaneous bacterial peritonitis, Others = influenza, acute kidney injury, pleural effusion, paroxysmal atrial fibrillation.

### 3.4 Factors associated with length of in-hospital stay

A multiple linear regression was calculated to predict the in-hospital stay based on the patients’ Child-Pugh class and groups according to the amount of ω-3 contained in the PN (control vs ω-3 enriched). A significant regression equation was calculated using presence of enhanced ω-3 and Child-Pugh class. Total bilirubin and MELD scores were not included due to competing risk with Child-Pugh class. The patients’ in-hospital stay was equal to 5.573 to 2.630 (Presence of enhanced ω-3) + 2.452 (Child-Pugh class). The patients’ in-hospital stay decreased 2.630 days when ω-3 enriched PN was given and increased 2.452 days for each increase in Child-Pugh class. Both presence of ω-3 enriched PN (*P* < .001), and low Child-Pugh score (*P* = .002) were significant predictors of reduction in-hospital stay (Table 4).

**Table 4:** Factors associated with in-hospital stay.

Dependent variable	Independent variable	B	β	VIF
In-hospital stay	Constant	5.573		
	Child-Pugh Class^∗^	2.452	0.256	1.001
	Use of ω-3^∗^	−2.630	−0.204	1.001

Adjusted R^2^ = 0.101, *P* < .001 (full model).

∗ Reference: Child-Pugh A: patients who received control parenteral nutrition.

## 4 Discussion

In this study, we demonstrate that acute variceal bleeding cirrhotic patients receiving ω-3 enriched PN after endoscopic variceal ligation have a shortened in-hospital stay compared to the control group. (7.9 days vs 10.7 days, *P* = .001).

The factors associated with the length of in-hospital stay were the Child-Pugh class of the patient and the use of ω-3 enriched PN. The result that an increase in Child-Pugh class lengthens the in-hospital stay is an established fact from numerous studies done in cirrhotic patients regarding the prognosis, morbidity, and mortality rates of the hospitalized patients.^[13,14]^ Therefore, the results of our study indicating that each increase in Child-Pugh class is associated with a 2.452 day increase in hospital stay is expected. It is also noteworthy that the baseline characteristics of the ω-3 enriched PN, and the control group showed no significant difference in the constitution of the Child-Pugh class in this study.

In direct comparison of the characteristics of the control group and the ω-3 enriched PN group, there were no significant differences in the comorbidities or cause of liver cirrhosis. The baseline systolic blood pressure of the ω-3 enriched PN group was significantly lower. Hypotension on initial presentation is generally associated with worse outcome in acute gastrointestinal bleeding patients as it reflects a greater loss of circulatory blood volume.^[15]^ Although the baseline systolic blood pressure of the ω-3 enriched PN group was significantly lower, the mortality rate of the ω-3 enriched PN group and the control group was not statistically different.

Gong et al^[16]^ report a decrease in total complication rate after hepatectomy in patients given ω-3 enriched PUFA-based PN. The difference in the liver-related morbidity, such as rebleeding, hepatic encephalopathy, spontaneous bacterial peritonitis and hepatic failure of the ω-3 enriched PN group and the control group was insignificant in this study. However, the morbidity rate not relating to the liver function was significantly lower in the ω-3 enriched PN group (1.2%) compared to the control group (7.4%) (*P* = .049). These findings may be attributed to ω-3 enriched PN modulating the immunity of the patients. Immunonutrition containing ω-3 enriched PUFA are administered to increase the nitrogen balance and protein synthesis, and improve the host immune state.^[17,18]^ As such, multiple societies including ASPEN and ESPEN recommended immunonutrition to all patients operated on for a digestive cancer 5 to 7 days prior to surgery whatever the patients’ nutritional status.^[19,20]^ Linecker et al^[21]^ recently stated that perioperative ω-3 failed to confer protection in patients undergoing hepatectomy in a multi-national randomized controlled trial. However, the heterogeneity in patient characteristics and surgical methods of each center was a major limitation of the study.

There have been concerns that EPA and DHA could increase the risk of bleeding by competing with arachidonic acid for incorporation into the platelet membrane or cyclooxygenase mediated pathways, leading to reduced production of arachidonic acid-derived prothrombotic metabolites (e.g., thromboxane A2) and increased production of antithrombotic EPA metabolites which could reduce platelet activation and aggregation.^[22]^ However, there was no increased risk of rebleeding or any other bleeding event in the ω-3 enriched PUFA PN group.

The novel finding of our study is that the ω-3 enriched PUFA-based PN had a significantly shorter in-hospital stay compared to patients in the control group in acute variceal bleeding cirrhotic patients. The result is comparable to studies done in cirrhotic patients undergoing hepatectomy due to liver cancer stating that the addition of ω-3 PUFA-based PN significantly decreased the length of hospital stay.^[16,23]^ A recent meta-analysis of patients undergoing hepatectomy also state that ω-3 PUFA administration has a positive impact on the liver function and inflammatory reaction.^[24]^

Greater energy intake is needed in acute variceal bleeding cirrhotic patients. However, recommendations to avoid oral and/or enteral nutrition in these patients for 48 hours may lead to inadequate energy supply.^[7]^ All the patients in our study were given PN to avoid hepatic glycogen depletion and protein catabolism in the acute stage of variceal bleeding. The favorable results of ω-3 PUFAs may be associated with the strong anti-inflammatory and immunomodulatory effects directed by replacing arachidonic acid as an eicosanoid precursor and altering the expression of inflammatory genes by influencing the transcription factor activation mechanisms.^[25]^

A key question to the results of the study was if 3 days of PN does have a significant effect in the morbidity, mortality, and disease course of the patient. A kinetic study in healthy volunteers revealed that a single oral dose of ω-3 PUFA is able to rapidly induce a shift in the ω-3 PUFA plasma profile within a few hours.^[26]^ Also, the short term administration of ω-3 PUFA just prior to ischemic insults was strongly protective in animal models.^[27]^ In addition, the studies included in the meta-analysis of patients undergoing hepatectomy stating the positive effect of ω-3 PUFAs administration reported 3 to 6 days of PN intervention.^[24]^ These findings support the validity of the beneficial effect of ω-3 enriched PUFA-based PN in acute variceal bleeding cirrhotic patients.

The retrospective design of the study is a major limitation of the study. However, the treatment protocol of our center, including medication and procedure methods, were implemented without much deviation unless clinically necessary. Only the PN types was the major difference in the 2 groups’ treatment protocol. Secondly, there is a deficiency in biomarkers that could explain the pathways leading to the beneficial effects of ω-3 enriched PUFA based PN.

Notwithstanding some of the limitations discussed above, this is the first study to establish that ω-3 enriched PUFA based PN in liver cirrhosis patients with acute variceal bleeding leads to shortened hospital stays. Further studies to determine the exact mechanisms leading to these beneficial effects are warranted.

## Author contributions

**Conception and design:** J.Y.C., S.J.K.

**Acquisition of data:** S.J.K., I.A.C., J.H.L.

**Analysis:** S.J.K., J.Y.C.

**Interpretation of the data and drafting of the article:** S.J.K., J.Y.C.

**Critical revision of the article for important intellectual content:** J.Y.C., J.H.L., J.L., Y.D.K., C.G.P.

Final approval of the article: all authors.
